# Morphotype analysis of the sibling vole (*Microtus rossiaemeridionalis*) casually introduced to the Russian Far East

**DOI:** 10.1007/s13364-012-0092-y

**Published:** 2012-09-01

**Authors:** Mikhail Petrovich Tiunov, Irina Vasiljevna Kartavtseva, Alexander Sergeevich Lapin

**Affiliations:** 1 Institute of Biology and Soil Science, Far East Branch, Russian Academy of Sciences, Prospekt 100-letya 159, 690022 Vladivostok, Russia; 2 Far Eastern State University of Humanities, 680000 Khabarovsk, Russia; 3 Khabarovsk Antiplague station, 680031 Khabarovsk, Russia

**Keywords:** *Microtus rossiaemeridionalis*, Far Eastern Russia, Teeth, Morphology, Voles

## Abstract

Here, we present the morphotypic variety of the m1 and M3 teeth diagnostics for the recently formed isolated population of the sibling vole in Far Eastern Russia. In the Far Eastern population, the prevalence of the individuals with m1 with a complicated crown of the forward unpaired loop of the paraconid is characteristic. Namely, m1 in these individuals shows well-expressed sixth exterior and fifth interior salient angles. The structure of the M3 morphotypes is also unique in the sibling voles in Far Eastern Russia. The dominant morphotypes were typica (47 %) and simplex (45 %), whereas the abundance of the duplicata morphotype was 0.08 %. The frequencies of various m1 and M3 morphotypes found in casually introduced sibling voles in the Far East are not typical of any previously studied *Microtus rossiaemeridionalis* population.

## Introduction

The original area of distribution of the sibling vole is located between 30–60° E and 60–40° N. Isolated sibling vole populations are found in the south of Russia, in Krasnoyarskij Kraj, Khakassia, and Irkutsk Oblast. In 2009–2010, a new isolated population of this species was discovered in the south of the Russian Far East in Khabarovskij Kraj in the vicinity of Sovetskaya Gavan City (N 48° 58′37.94″, E 140° 13′15.90″) (Kartavtseva et al. 2011, 2012). This population is at the final point of the Bajkal-Amur Mainline, 4,200 km from Irkutsk City. It is obvious that the local sibling voles, as well as the voles from other isolated populations located along the railway, were casually introduced by humans (Kovalskaya and Malygin 1985).

It is obvious that the translocation of a limited number of individuals includes the island mechanisms of microevolutionary processes, including geographical isolation, a random sample of allelic frequencies, and inbreeding. At the same time, the possibility of the future formation of a new morphological form of the introduced vole in isolated habitats depends in many respects on the environmental conditions in the new habitats. It is generally supposed that if no considerable environmental changes occur in a habitat, the heterogeneity of the initial population supports the genetic “memory” and interferes with the emergence of new morphological forms (Korablev et al. 2010). The detection and monitoring of trends in microevolutionary processes in new habitats will be most accurate if these investigations are based on the early documentation of the phenetic and morphological characteristics of the introduced vole. Accordingly, this work aims to characterize the morphotypic variety of the m1 and M3 teeth. These teeth are relatively prone to change and are used as diagnostics for the recently formed isolated population of the sibling vole from the Far Eastern Russia in comparison with European and the Siberian populations.

## Material and methods

**Fig. 1:**
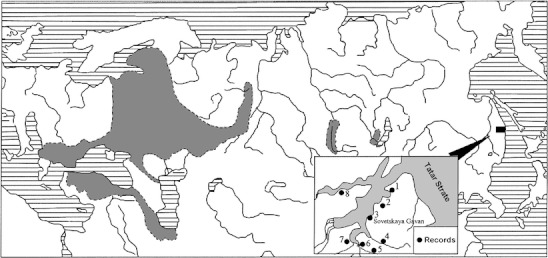
The range of *M. rossiaemeridionalis* Ognev, 1924 (according to Shenbrot and Krasnov 2005) and capture sites in the Russian Far East: (*1*) Lososin rural settlement, (*2*) outskirts of Lososin rural settlement, (*3*) Sovetskaya Gavan City, Bunker port territory, (*4*) Bolshaya Okocha River, (*5*) Malaya Egge River, (*6*) left bank of lower reaches of the Bolshaya Egge River, (*7*) bank of Bukhta Egge, (*8*) northeast outskirts of Mayskii rural settlement

We analyzed 66 right and left lower (m1) and 64 right and left upper molars (M3) belonging to 33 individuals caught in Khabarovsky Krai, in urbanized biotopes in the vicinities of Sovetskaya Gavan City and two nearby settlements (Fig. 1). The species was identified by karyological characteristics—2*n* = 54, NF = 56, and C-banding—and morphological characteristics—body, cranium, baculum, and spermatozoa (Kartavtseva et al. 2012).

When analyzing dental variation in hypselodont arvicolines, the common practice is to partition the samples into the groups of juvenile and non-juvenile individuals based on the presence or absence of the juvenile characters such as juvenile folding at m1 and M3 (individuals with incompletely formed of the occlusal surface) (Nadachowski 1982; Borodin 2009). All individuals studied exhibited non-juvenile patterns of tooth wear on all molars including M3.

In this study, we focused primarily on qualitative traits corresponding to the development of additional salient angles on the lingual or/and on buccal side of a tooth. For m1, we used the classification proposed by Markova et al. (2010), including four morphotypes. Membership in a certain morphotype depends on the extent of complication of the enamel contour of the anterior unpaired loop. Morphotype I is characterized by a trifolium-like anterior loop without crown complication or with a small enamel prominence on the exterior part of the crown, morphotype II has a distinctly expressed sixth lingual salient angle, morphotype III has a distinctly expressed fifth buccal salient angle, and morphotype IV has both a distinctly expressed sixth lingual salient angle and a fifth buccal salient angle. For M3, the traditional classification proposed by Rörig and Börner (1905) includes such morphotypes as *simplex* (s), *typica* (t), *duplicata* (d), and *variabilis* (v). The simplex morphotype is defined by interior and exterior tooth sides with three salient angles each. The typica morphotype is defined by four salient angles at the interior edge and three salient angles at the exterior edge. The duplicata morphotype is defined by four salient angles at each edge. The variabilis morphotype is defined by four to five salient angles at the interior edge and five to three or four salient angles at the exterior edge.

## Results and discussion

Basic morphological and craniological characteristics show that the *Microtus rossiaemeridionalis* found in the Far East do not surpass the variability limits of the species. The following averaged measurements characterize the Far Eastern individuals. All measurements were made in millimeters. The body length is 97.6 ± 9.2 (*n* = 18), the tail length is 35.2 ± 3.5 (*n* = 16), the hind foot length is 15.4 ± 1.4 (*n* = 18), and the ear length is 11.2 ± 1.0 (*n* = 18). The condylobasal length is 23.3 ± 1.5 (*n* = 17), the length of the upper teeth is 5.6 ± 0.4 (*n* = 16), the length of m1 is 2.65 ± 0.10 (*n* = 16), and the length of M3 is 1.78 ± 0.09 (*n* = 16). All individuals studied have six plantar calluses.

**Fig. 2:**
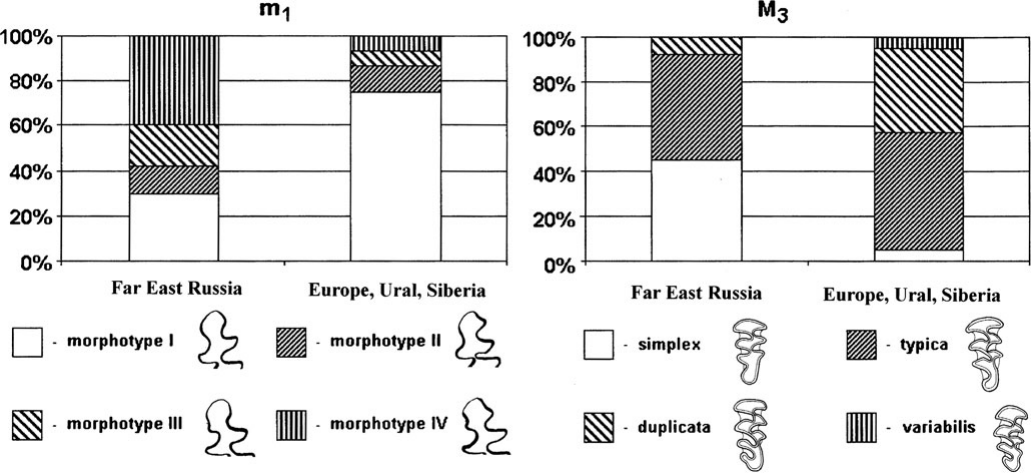
Average frequencies of main molar morphotypes in *M. rossiaemeridionalis*. See “Materials and methods” for morphotype abbreviations

In *M. rossiaemeridionalis*, morphotype I of m1 and typica of M3 are predominant in all samples (Malygin 1978; Meyer et al. 1996; Markova et al. 2010). The duplicata form is also present in all samples, although it varies in frequency from 10 to 65 % in *M. rossiaemeridionalis* (Markova et al. 2010). In contrast, in the Far Eastern population, the prevalence of the individuals with m1 with a complicated crown of the forward unpaired loop of the anteroconid (morphotypes III–IV) is characteristic. These morphotypes amount to 70 %. The majority of these individuals belong to morphotype IV (40 %) (Fig. 2), i.e., m1 in these individuals shows well-expressed BSA6 and LSA5 (buccal and lingual salient angles). This pattern of m1 morphotype frequencies is not known to occur in any other *M. rossiaemeridionalis* populations (Markova et al. 2010).

Note that different morphotypes at right and left molars is not prevalent in the Far Eastern population. Only four instances of asymmetry in M3 were found in the 33 individuals studied. Three individuals show two different morphotypes—simplex and typica (two animals) and typica and duplicata (one animal).

The structure of the M3 morphotypes is likewise unique in the sibling voles in Far Eastern Russia. Only three M3 morphotypes have been found in these populations: simplex, typica, and duplicata. The rare variabilis variant observed in a number of sibling vole populations was not found in the animals in this study. The dominant morphotypes were typica (47 %) and simplex (45 %), whereas the abundance of the duplicata morphotype was 8 % (Fig. 2). A similar pattern was observed only in sibling voles from Moldova, where the typica frequency was 5 %, the simplex frequency was 33 %, and the duplicata frequency was 17 % (Markova et al. 2010). However, as is the case in other populations of sibling voles, the predominant individuals were of the simplest (m1) morphotype. The frequencies of various m1 and M3 morphotypes found in casually introduced sibling voles in the Far East are not typical of any previously studied *M. rossiaemeridionalis* populations, including ten populations from Europe (338 m1/329 M3), two populations from Middle Urals (180 m1/180 M3), three populations from Southern Urals (62 m1/44 M3), and one population from Siberia (30 m1/30 M3) (Markova et al. 2010).

*M. rossiaemeridionalis* easily enters man-made structures and reaches high population numbers in urbanized areas (Tikhonov et al. 1992, 1998, 1997, 2001; Karaseva et al. 1994). This vole is characterized by a significant ability to survive and to adapt to new conditions (Fredga et al. 1990; Frafjord 2002). It is obvious that the introduction of the sibling vole to the Far East became possible only after 1945, when the railway traffic to Sovetskaya Gavan City began. It is probable that the introduction occurred after the completion of the railway bridge over the Amur River in 1975. In any case, it would be too early to explain all novel morphological characteristics of the sibling vole in the Far East in terms of microevolutionary processes. Nevertheless, it is certain that the conditions under which the sibling vole lives in the Far East (a monsoon climate with a mild, snowy winter, and a cool, rainy summer) naturally differ from the conditions in the typical sibling vole habitats of Eastern Europe and the Ural Mountains. In the Far East and throughout most of its range, the sibling vole inhabits herb meadows, fallow lands, and urbanized zones. Moreover, it should be noted that individuals of *M. rossiaemeridionalis* have been collected near the tidal zone in the vicinity of the port facilities in the settlement of Lososina.

It is still not possible to infer the area of origin of the introduced populations of *M. rossiaemeridionalis*. Morphotype analyses of m1 and M3 exist only for series of selected areas of the entire range of this species (Markova et al. 2010), and none of them is similar to the Far Eastern population. Moreover, we do not know the dental characteristics of the earliest introduced individuals, which might be different from the data presented in this paper.
